# Identification and validation of reference genes for quantitative real-time PCR studies in long yellow daylily, *Hemerocallis citrina* Borani

**DOI:** 10.1371/journal.pone.0174933

**Published:** 2017-03-31

**Authors:** Feifan Hou, Sen Li, Jinyao Wang, Xiuping Kang, Yiqun Weng, Guoming Xing

**Affiliations:** 1 Horticulture College, Shanxi Agricultural University, Taigu, China; 2 USDA-ARS Vegetable Crops Research Unit, Horticulture Department, University of Wisconsin—Madison, Wisconsin, United States of America; Huazhong Agriculture University, CHINA

## Abstract

Gene expression analysis using reverse transcription quantitative real-time PCR (RT-qPCR) requires the use of reference gene(s) in the target species. The long yellow daylily, *Hemerocallis citrina* Baroni. is rich in beneficial secondary metabolites and is considered as a functional vegetable. It is widely cultivated and consumed in East Asian countries. However, reference genes for use in RT-qPCR in *H*. *citrina* are not available. In the present study, six potential reference genes, *actin* (*ACT*), *AP-4 complex subunit* (*AP4*), *tubulin* (*TUB*), *ubiquitin* (*UBQ*), *18S* and *60S ribosomal RNA*, were selected and their expression stability in different developmental stages, organs and accessions was evaluated using four statistical software packages (geNorm, NormFinder, BestKeeper, and RefFinder). For commercial flower buds of different landraces, the combination of *60S*, *TUB*, and *AP4* was appropriate whereas *ACT* and *60S* was suitable for normalization of different organs. In addition, *AP4* exhibited the most stable expression in flower buds among different developmental stages. *UBQ* was less stable than the other reference genes under the experimental conditions except under different organs was *18S*. The relative expression levels of two genes, *primary-amine oxidase* (*HcAOC3*) and *tyrosine aminotransferase* (*HcTAT*) which play important roles in alkaloid biosynthesis were also examined in different organs of the ‘Datong’ landrace, which further confirmed the results of selected reference genes. This is the first report to evaluate the stability of reference genes in the long yellow daylily that can serve as a foundation for RT-qPCR analysis of gene expression in this species.

## Introduction

The long yellow daylily (LYD hereinafter), or yellow flower vegetable (huang hua cai in Chinese), *Hemerocallis citrina* Baroni., is a perennial herb in the family Liliaceae, which is native to central and northern China, the Korea Peninsula, and Japan [1]. LYD is widely cultivated in this region and used as an ornamental plant, vegetable or medicinal plant because of its beautiful flower, pleasant flavor, and beneficial secondary metabolites; thus it is also considered as a functional vegetable crop [2]. The consumed part of this plant is mainly its flower buds, which are harvested before flowering. The LYD returns green in March, blooms from June to September in the summer. This crop is easily adaptable to various growth environments.

The alabastrums of LYD are rich in rutin, hesperidin, and colchicine. These secondary metabolites are used to treat anxiety and swelling [3, 4]; they also have their applications in modern medicine [5]. The chemosynthesis and pharmacology of secondary metabolites from plants have been extensively investigated in a number of plant species, but with no report in LYD so far. Molecular biology methods have been used to study the biosynthetic pathway of secondary metabolites and to examine functional genes in plants [6]. These approaches can be used to identify the metabolic pathways of active constituents in plants and to determine functional genes and their expression patterns in these pathways [7].

Reverse transcription quantitative real-time PCR (RT-qPCR) is widely used to analyze relative gene expression abundance in living organisms. Many factors influence the reliability of the results including RNA quality, expression levels of target genes and other factors that contribute to non-uniform test results [8, 9]. To obtain the true differences in the expression levels of target genes by RT-qPCR, stably expressed reference genes are used as internal controls for standard correction [10, 11]. It is difficult to find ideal reference genes that are stable under diverse experimental conditions. The expression of reference genes may vary depending on plant developmental stages, organs, varieties and physiological conditions [12]. For example, in *Lilium brownii*, *EF1α* and *18S rRNA* were the most stable reference genes for total samples, while *psaA* and *EF1α* were optimally stable in stressed root tissues [13]. For *Hedera helix*, *F-box* gene was more stable than other examined genes under all analysis conditions, except under abscisic acid (ABA) treatment for which *40S rRNA* was the most stable reference gene [14]. Thus, it seems there are no universal reference genes, and the selection has to be conducted in individual species.

In the present study, the expression stability of six commonly used candidate reference genes (*18S rRNA*, *60S rRNA*, *UBQ*, *AP4*, *ACT*, and *TUB*) were examined in different growth stages, organs and landraces of LYD. The expression stability of these reference genes was analyzed by geNorm, NormFinder, and BestKeeper. RefFinder was used to integrate information and obtain a comprehensive ranking of the six candidate reference genes. In addition, the expression levels of two target genes in LYD, *HcAOC3* (*primary-amine oxidase*) [15] and *HcTAT* (*tyrosine aminotransferase*) [16–18] that play roles in biosynthesis of alkaloids, were used to verify the reliability of the selected reference genes. The results showed that *AP4*, the combination of *60S*, *TUB*, *AP4*, or *ACT* along with *60S* was suitable for normalization in different developmental stages, landraces, and organs of LYD, respectively.

## Materials and methods

### Plant materials and treatments

**Fig 1:**
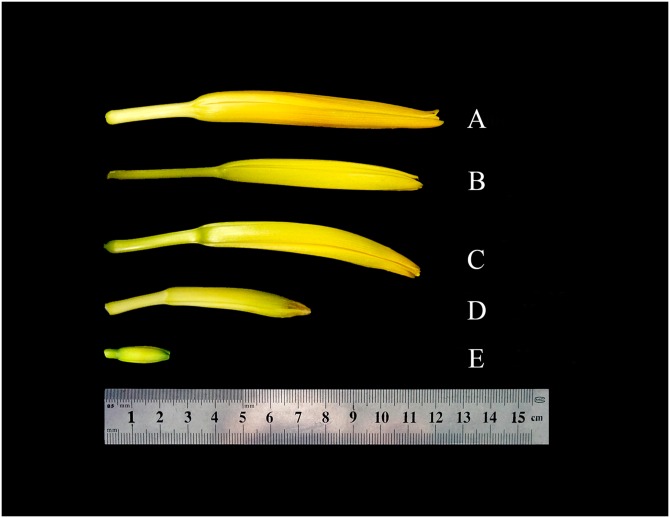
Commercial stage flower buds of different landraces and flower buds at different developmental stages of ‘Datong’. A, B and C are commercial-stage flower buds of ‘Panlong’, ‘Changzuizi’ and ‘Datong’ landraces, respectively. D and E are flower buds of ‘Datong’ landrace at 0-5cm and 5-10cm stage, respectively.

Three LYD accessions, ‘Datong’, ‘Panlong’, and ‘Changzuizi’ were used in the present study. All of them were perennial landraces that were grown from tuber with buds since 2011. Plants were maintained in the *Hemerocallis* germplasm nursery at Shanxi Agricultural University, Taigu, China, where the summer average temperature is 22.8°C and rainfall concentrates in July to September. We investigated expression of six candidate reference genes in the ‘Datong’ landrace at three development stages based on the length of flower bud: <5 cm, 5–10 cm and >10 cm (the maximum length of the flower bud of ‘Datong’ was 15 cm before anthesis). We compared organ-specific expression of these genes in the root, leaf, and flower bud (>10 cm) tissues of ‘Datong’ plants which were all collected at florescence. Finally, we also compared expression of these genes in the flower buds at commercial harvest stage (the day before flowering) from ‘Changzuizi,’ ‘Panlong,’ and ‘Datong’ landraces (Fig 1, S1 Fig). In all treatments, there were three biological replicates for each sample. The collected samples were immediately frozen in liquid nitrogen and stored at -80°C for RNA extraction.

### RNA isolation and cDNA synthesis

Total RNA was extracted using an RNAprep Pure Plant Kit (Tiangen, Beijing, China) according to manufacturer’s protocols. The RNA concentration and purity were determined with a NanoDrop 2000c Spectrophotometer (Thermo Fisher Scientific Inc., Waltham, MA, USA). The 260/280 nm ratio was detected to be between 1.9 and 2.1. The RNA integrity was also confirmed on 1% agarose gel electrophoresis. Total RNA (0.5 μg) was used for reverse transcription with the PrimeScript RT Reagent Kit (Takara Bio Inc., Kusatsu, Japan) in a 20-μL reaction volume according to the manufacturer's manual.

### Primer design and RT-qPCR conditions

We conducted RNA-Seq of the ‘Datong’ LYD transcriptome. The cDNA samples from root, leaf, and flower bud of the ‘Datong’ landrace were sequenced with Illumina HiSeq^™^ 2500 (Biomarker, Beijing, China) and an EST contig assembly was developed using homologous sequences from lily and garlic, both of which are also in the family Liliaceae (unpublished data). Primer sequences for all six candidate reference genes, *18S rRNA*, *60S rRNA*, *UBQ*, *AP4*, *ACT*, and *TUB* from previous studies [19–21] were used to BLAST the ‘Datong’ EST contig assembly to obtain corresponding homologous gene sequences in the LYD genome. New primers for these genes were designed using the Primer Premier 5 software (PREMIER Biosoft, Palo Alto, CA, USA) with the following criteria: primer length of 17–25 nucleotides, expected amplicon lengths of 80–150 bp, melting temperature (Tm) of 58–62°C, and GC content of 45–55%. All primers were synthesized commercially (Zoonbio Biotechnology, Nanjing, China) and tested by regular PCR. The amplicons were analyzed by 1.5% agarose gel electrophoresis before RT-qPCR. Standard curves (S2 Fig) using 5-fold dilution series of pooled cDNA were developed to calculate the PCR amplification efficiency (E) and the regression coefficient (*R*^*2*^) of each primer pair [12]. For all six candidate reference genes, their species sources and GenBank accession numbers of homologous genes, primer sequences, amplicon length, Tm (melting temperature), E (PCR amplification efficiency), and *R*^2^ (regression coefficient of standard curve) are listed in Table 1.

**Table 1:** Candidate reference genes descriptions and primer sequences.

Gene	species source	GeneBank accession #	Primer sequences(forward/reverse)	amplicon length(bp)	Amplicon Tm(°C)	E(%)*	R^2^*
18S	*Lilium formosanum*	D29775	CAGACAAATCGCTCCACCAAC	200	59.0	98.7	0.999
			CGCAAGGCTGAAACTTAAAGG				
60S	*Lilium davidii* var. unicolor	KP861879	CGTCTTCCTATTCGCCAACC	90	60.9	101.2	0.997
			AGCACCGCCAAAGTCCAGTT				
UBQ	*Lilium longiflorum*	AF116772	CAGTAATGGCGATCAAAGTGG	86	58.8	102.3	0.998
			AAGGTGGTCAGGCTCAGGAA				
AP4	*Lilium davidii* var. unicolor	KP861878	ATTTCCTCCCTCTTCCTACCC	105	59.4	99.7	0.998
			TGGACCTGCTGCGATGTTTAT				
ACT	*Lilium regale*	JX826390	GCAAGGAAATCACGGCACT	91	58.9	101.1	0.999
			GAACCTCCAATCCAAACACTGTAC				
TUB	*Allium sativum*	KP116310	CTTGAACCGGGTACGATGGA	110	60.0	97.5	0.999
			CCCTTGGCCCAATTATTCC				

*E = PCR amplification efficiency of candidate reference genes.

*R^2^ = regression coefficient of standard curve for each candidate reference genes.

RT-qPCR was carried out with the ABI prism 7500 Fast Real-time PCR system (Thermo Fisher Scientific Inc., Waltham, MA, USA) with the SYBR Green chemistry. Each reaction was performed in a 20-μL volume including the following components: 1 μL diluted cDNA template (0.1 mg. mL^-1^), 10 μL SYBR Premix Ex Taq II (Takara Bio Inc.), 0.4 μL of ROX Reference Dye II (Takara Bio Inc.), 0.8 μL each of forward and reverse primers (10 μM), and 7 μL ddH_2_O. The reaction conditions were as follows: 95°C for 3 min, followed by 40 cycles of denaturation at 95°C for 30 s and annealing at 55°C for 30 s, and extension at 72°C for 1 min. The melting curve was obtained by heating the amplicon from 55°C to 95°C at increments of 0.4°C for 30 s. Each RT-qPCR analysis was performed with three technical replicates.

### Assessment of reference gene expression stability

Three common statistical software programs, geNorm [22], NormFinder [23], and BestKeeper [24], were employed to calculate and analyze the expression stability of six reference genes across different experimental samples. The comprehensive ranking of the stability of six reference genes was determined using RefFinder [25].

Raw RT-qPCR data were obtained using ABI prism 7500 software V2.3 (Thermo Fisher Scientific Inc.), and the cycle threshold (Ct) values were used to analyze the expression levels of candidate reference genes. For geNorm and NormFinder, raw Ct values were transformed into ΔCt values, which have a maximum value of 0, while all other values are negative before calculation. The value of 2^(ΔCt)^ was calculated for every data point, and all data were assessed relative to the expression of the highest expressed data point. For geNorm, the expression stability value (M) of each reference gene was calculated based on the average pairwise variation among all tested genes; a lower M value represents higher gene expression stability [22]. For NormFinder, an ANOVA-based model of each reference gene was used to calculate the expression stability value by determining inter- and intra-group variation; in this analysis, the gene with the lowest value has the most stable expression [23].

The BestKeeper software program uses raw Ct values to calculate the stability of candidate reference genes. The expression stability of reference genes was analyzed using BestKeeper software based on the following three parameters: the standard deviation (SD), the percentage of covariance, and the correlation coefficient (*r*) [24]. Genes with SD values greater than 1 were considered unreliable for expression normalization. Reference genes with lower SD values are more stable and therefore more suitable for RT-qPCR [26].

The comprehensive ranking of reference genes was obtained by RefFinder using raw Ct values. Based on the rankings produced by other three programs, it assigns an appropriate weight to each gene and calculates the geometric mean of their weights to produce an overall final ranking [25]. The most stable LYD reference genes were then determined from the different experimental samples.

### Validation of reference gene analysis

*AOC3* and *TAT* are key enzyme-coding genes in the biosynthesis of alkaloids. Our transcriptome sequencing database showed that these two genes, designated as *HcAOC3* and *HcTAT* (the sequencing data were presented in S1 Text), respectively hereinafter, have high relative expression levels in roots of LYD (unpublished data). They were used as target genes to verify the stability of the reference genes for RT-qPCR. Through BLAST search, we identified the whole length cDNA sequences from ‘Datong’ transcriptome contig assembly. The relative expression levels of *HcAOC3* and *HcTAT* in different organs of ‘Datong’ landrace plants were determined and normalized with the most and least stable reference genes according to geNorm, NormFinder, BestKeeper, and RefFinder results. Three biological replicates and technical replicates were adopted for each treatment. The leaf was used as the control sample. The cycle threshold (Ct) value of reference gene was subtracted from that of target gene to obtain a ΔCt value. The ΔCt value of leaf was subtracted from the ΔCt value of root or flower to obtain a ΔΔCt value. The fold changes in relative expression level to leaf were expressed as 2^−ΔΔCT^[27]. The *t*-test statistics were generated from an ANOVA model by SASS program and characterized among-relative expression levels in the same organ sample when different reference genes were used. Primers design and qRT-PCR reactions were followed as mentioned before. The following primer pairs were used for RT-qPCR: 5′-CGTGACCCAAAGGTTGTGCT-3′ (forward) and 5′-TGTTCCTGGTTCTAACTGCCTAC-3′ (reverse) for *HcAOC3* and 5′-TTGGAGACTTGGGTGGATGG-3′ (forward) and 5′-TGAGGAACTGCTGCCTGAATAA-3′ (reverse) for *HcTAT*.

## Results

### Verification of PCR amplicons and primer specificity

Agarose gel electrophoresis was used to examine the size of PCR amplicons, thereby ensuring that all primer pairs amplified fragments of the expected size (S3 Fig). Melting curve analysis also indicated that each primer pair amplified products corresponding to a single fragment (S4 Fig). As shown in Table 1, the amplification efficiencies (E) of RT-qPCR among all six reference genes varied from 97.5% for *TUB* to 102.3% for *UBQ*; the regression coefficients (*R*^*2*^) ranged from 0.997 for *60S* to 0.999 for *18S*, *ACT* and *TUB*.

### Expression profile of candidate reference genes

**Fig 2:**
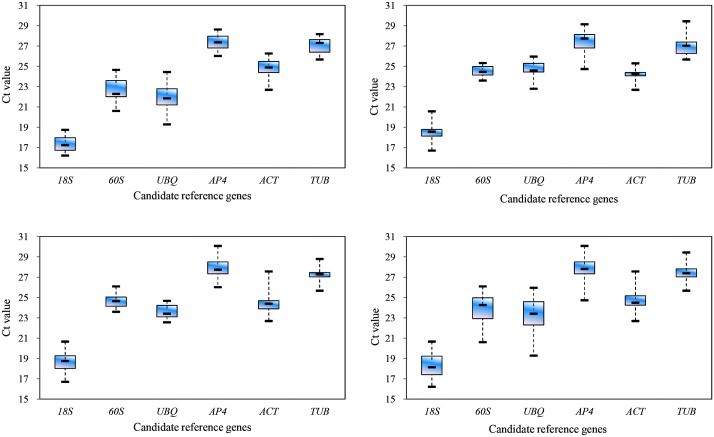
Box-whiskers plot showing Ct variation of six candidate reference genes in the LYD samples. (A) flower buds at different developmental stages of ‘Datong’ landrace; (B) different organs of ‘Datong’ landrace; (C) commercial flower buds of different landraces; and (D) all samples. The horizontal line within each box represents the median value. For each box, the upper and lower edges indicate the 25th and 75th percentiles, while whiskers represent the maximum and minimum values.

The Ct values of the six candidate reference genes detected by RT-qPCR are presented in Fig 2 with lower Ct value indicating higher expression level. Among the six genes, *18S* had the lowest Ct value across all samples. The six genes can be divided into two groups; group 1 including *18S*, *60S*, and *UBQ* with mean Ct values below 23 cycles suggesting relatively high expression levels; group 2 including *ACT*, *AP4*, and *TUB* with mean Ct values higher than 23 cycles indicating relatively low expression levels. The variation ranges (lower values represent less variability) of the six reference genes differed within experimental factors (Fig 2). For flower buds at different developmental stages, *18S* had the least variation (2.53 cycles), whereas *UBQ* had the most (5.15 cycles). Among the different organs, *60S* had the narrowest range of variation (1.73 cycles) while *AP4* had the widest range of variation (4.4 cycles). Among the commercial flower buds of different landraces, *UBQ* showed the least variation (2.11 cycles) and *ACT* showed the highest (4.88 cycles). Likewise, *TUB* was the most stable (3.76 cycles) while *UBQ* was the least (6.68 cycles) across all samples from three different experimental treatments.

### Expression stability of all candidate reference genes

The raw Ct values of the six candidate reference genes exhibited a wide range of variations and did not represent the stability of genes accurately. Thus, it was necessary to use other statistical approaches for evaluating the results. As such, four programs (geNorm, NormFinder, BestKeeper, and RefFinder) were used to determine the stability of the six candidate reference genes.

For geNorm, an M value below the threshold of 1.5 was used to identify reference genes as stably expressed. This was calculated using the average pairwise variation among all genes tested and subsequently ranking them according to the stepwise exclusion of the least stable gene. M values of all the tested reference genes in the samples from the different treatments are presented in Table 2 which were all lower than 1.5. *TUB* and *UBQ*, respectively, were the most and least stable genes among flower buds at different developmental stages, commercial flower buds of different landraces, and all samples from three different experimental treatments. Other genes with highly stable expression were *AP4* for flower buds at different developmental stages, *60S* and *ACT* among different organs, and *60S* for commercial flower buds of different landraces. Among different organs, *18S* was the least stable gene. When all samples were considered together, *ACT* exhibited the most stable expression.

**Table 2:** Average expression stability values (M) for the six candidate reference genes in the LYD samples as calculated using the geNorm algorithm.

Among flower buds at different developmental stages (‘Datong’)		Among different organs (‘Datong’)		Among commercial flower buds of different landraces		All samples	
Ranking	M*	Ranking	M	Ranking	M	Ranking	M
AP4	0.46	60S	0.45	60S	0.38	TUB	0.77
TUB	0.46	ACT	0.45	TUB	0.38	ACT	0.77
ACT	0.56	UBQ	0.47	AP4	0.52	AP4	0.83
18S	0.89	TUB	0.69	ACT	0.56	18S	1.13
60S	1.19	AP4	0.85	18S	0.72	60S	1.26
UBQ	1.38	18S	0.97	UBQ	0.83	UBQ	1.40

*M = stability value.

**Fig 3:**
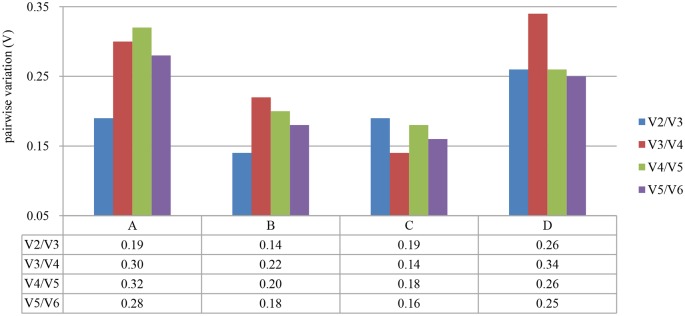
Pairwise Variation (V) analyses of six candidate reference genes in LYD samples. Pairwise variation (V_n_/V_n+1_) values were analyzed using the geNorm program. (A) flower buds at different developmental stages of ‘Datong’ landrace; (B) different organs of ‘Datong’ landrace; (C) commercial flower buds of different landraces; and (D) all samples. When the pairwise variation (V_n_/V_n+1_) is less than 0.15, it is recommended that no additional genes are required for the normalization.

The pairwise variation (V_n_/V_n+1_) was used to determine the optimal number of reference genes for normalization that were calculated by geNorm. The values were proposed to be less than 0.15 [28]. As shown in Fig 3, the pairwise variation of reference genes yielded a V_2_/V_3_ of 0.14 for samples from different organs and V_3_/V_4_ of 0.14 for samples from the commercial flower buds of different landraces. Therefore, *60S* and *ACT* could be considered the most stable reference gene combination among the different organs; similarly, *60S*, *TUB*, and *AP4* was the best reference gene combination for achieving stable expression among the commercial flower buds of different landraces. However, all pairwise variation values of the reference genes for flower buds at different developmental stages and all samples were greater than 0.15. This indicated that at least five or more reference genes were needed for normalization of these two experimental conditions. But adding too many reference genes will increase the instability, and also the complexity of the experimental work [29]. Consequently, only one reference gene will be proposed to apply, resulting in accurate normalization.

The NormFinder analysis is based on an ANOVA model that considers intra- and inter-group variation to evaluate expression stability. The stability values of the six candidate reference genes were calculated by NormFinder under different experimental treatments (S1 Table). The NormFinder results were similar to those of geNorm. Expression levels of *AP4* among flower buds at different developmental stages, *ACT* among different organs, and *60S* among commercial flower buds of different landraces were the most stable reference genes with values of 0.18, 0.04, and 0.06, respectively. The maximum values for the stability of gene expression were for *60S* (0.28), *18S* (0.14), and *UBQ* (0.19). Among all samples, *AP4* and *18S* were the most and least stable reference genes, respectively (with values of 0.11 and 0.18). Additionally, *UBQ* (0.14) was more stable among all samples with NormFinder compared to the corresponding result obtained with geNorm.

The expression stability of the six candidate reference genes as analyzed using BestKeeper software indicated that *18S* (0.64) and *UBQ* (1.23) were the most and least stable reference genes for flower buds at different developmental stages, respectively (S2 Table). Among different organs, *ACT* had the most stable expression (0.43) whereas AP4 had the least (1.03). Among commercial flower buds from different landraces, *60S* (0.53) and *ACT* (0.84) showed the most and least stable expression, respectively. Among all three experimental treatments, *TUB* (0.62) was the most stable reference gene and *UBQ* (1.41) was the least stable reference gene, in accord with the resulted produced by geNorm.

The six candidate reference genes tested under the same condition were ranked using the analyses of the three statistical algorithms. The three programs inferred parallel trends, but there were some subtle differences in the specific rankings under each experimental condition. The discrepancies in rankings by different software for all samples were obvious; *AP4* and *18S* were, respectively the most and least stable reference gene using NormFinder, while the two genes exhibited intermediate stability for normalization according to geNorm and BestKeeper.

RefFinder was used to assess the overall aforementioned results and determine the comprehensive rankings of the six candidate reference genes (Table 3). Its algorithm integrates information from geNorm, NormFinder, and BestKeeper to compare and rank the tested candidate reference genes. Lower geometric means of the ranking values represent more suitable and stable reference gene. The analysis by RefFinder revealed that *AP4*, *ACT*, and *60S* exhibited the most stable expression among flower buds at different developmental stages (1.41), among different organs (1.00), and among commercial flower buds (1.00), respectively. However, *UBQ* and *18S* exhibited the least stable expression according to RT-qPCR results for samples under different experimental conditions. Among all samples, *TUB* was the most stable according to the comprehensive ranking, while *UBQ* was the least.

**Table 3:** Comprehensive rankings of the six candidate reference genes in the LYD samples based on the RefFinder software program.

Among flower buds at different developmental stages (‘Datong’)		Among different organs (‘Datong’)		Among commercial flower buds of different landraces		All samples	
Ranking	G*	Ranking	G	Ranking	G	Ranking	G
AP4	1.41	ACT	1.00	60S	1.00	TUB	1.00
18S	1.86	60S	1.68	TUB	1.68	ACT	2.66
TUB	2.06	UBQ	3.00	AP4	3.22	AP4	2.71
ACT	3.94	TUB	4.23	ACT	4.43	18S	3.13
60S	4.73	AP4	5.23	18S	5.00	60S	4.47
UBQ	6.00	18S	5.42	UBQ	5.05	UBQ	6.00

*A lower geometric mean (geomean) of the ranking values represents more expression stability of the reference genes. G = Geomean of ranking values.

### Reference gene validation

**Fig 4:**
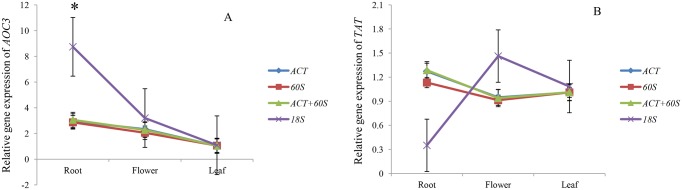
Relative expression level of (A) *HcAOC3* and (B) *HcTAT* in different organs of ‘Datong’ landrace plants. The most or the least stable reference genes were used for analysis. The error bars represent standard errors, and *t*-test statistics were generated from an ANOVA model characterizing among-relative expression levels in the same sample. * *P* < 0.05.

*HcAOC3* and *HcTAT* are two key genes encoding enzymes in the alkaloid biosynthesis pathway, which may be related to alkaloid metabolism in LYD. Their relative expression among different organs in the ‘Datong’ landrace was used to evaluate and normalize the results obtained by the four statistical programs (Fig 4). Based on results from geNorm and RefFinder, two reference genes were suitable for normalization of different organs. So, four sets of reference genes were selected: the most stable reference genes (*ACT*, *60S*, *ACT*+*60S*) and the least stable reference gene (*18S*) among different organs. As shown in Fig 4, when the most stable reference genes (*ACT*, *60S*, and *ACT*+*60S*) were used for normalization, there was no significant difference in relative expression of *HcAOC3* and *HcTAT* among different organs. The most stably expressed reference genes exhibited similar expression levels. However, when *18S* was used as a reference gene, there was significant difference among the relative expression level in root (*p* < 0.05); and the differences of relative expression of *HcTAT* between organs were not in according with the situation when the most stable reference genes (*ACT*, *60S*, and *ACT*+*60S*) were used as reference genes. The relative expression level of *HcTAT* in root below the level in flower, this was not in conformity with the previous results.

## Discussion

RT-qPCR is one of the most common molecular biology research tools, and its reliability and accuracy strongly depends on appropriate normalization using stably expressed reference genes [30]. Although several reference genes have been proposed for two Liliaceae species *Lilium brownie* [13] and *Lilium davidii* var. *unicolor* [20], their usefulness in LYD was not known. Reference genes suitable for one species are not necessarily working for others, and there are no one-fit-all reference genes with constantly stable expression across all plants species [31]. Our results indicate that the expression stability of reference genes was affected by developmental stages, tissues, landraces, and physiological status, which emphasizes the necessity of selecting suitable reference genes for RT-qPCR.

Raw Ct values are the foundation for evaluating reference genes [32]. They directly reflect the expression levels of genes, to a certain degree [33]. Many studies have reported that the transcript levels of reference genes may affect the quantification of target genes in RT-qPCR experiments. The selected reference gene should have similar Ct values to those of the target genes. In our study, *TUB*, *AP4*, *ACT*, and *60S* had moderate Ct values (varying from 20 to 30) under all experimental treatments and thus meet the requirement for use as reference genes.

The software packages geNorm, NormFinder, and BestKeeper are three of the most widely used statistical software programs for evaluating reference genes, and they use different algorithms. The results obtained using the three programs were largely consistent, despite subtle differences owing to the various statistical algorithms they employ. Such subtle discrepancies were also observed in previous studies. In kiwifruit, BestKeeper showed that *ACT1* was one of the most stable reference genes, whereas geNorm indicated that *ACT1* was among the least stable [34]. Therefore, it is helpful to evaluate the results with multiple programs to obtain integrated results for candidate reference genes.

Information based on pairwise variation values calculated by geNorm is also useful. In this study, two and three reference genes were proposed for normalization in different landraces and organs of LYD. Due to the various experimental treatments, and number or type of tested candidate reference genes, the optimal number of reference genes is different. Considering that the variation in the average of multiple reference genes was smaller than the variation in individual reference gene, the normalization calculation based on the geometric mean of multiple reference genes provided more accurate and reliable normalization of expression data [35]. This situation were common in other plants, such as *Lilium davidii* var. *unicolor* [20], watermelon [36], *Daucus carota* [37] and melon [38]. Although a value of less than 0.15 was proposed by this program, it should not be considered the must-follow criterion in selecting the optimal number of reference genes for RT-qPCR [39].The balance between cost and accuracy of analysis should be taken into consideration [40]. Also, we used RefFinder to integrate results from geNorm, NormFinder, and BestKeeper. This program assigns an appropriate weight to tested genes and calculates the geometric mean of their weights using ranking information from the three programs. The comprehensive ranking by RefFinder made the selection of candidate reference genes reliable [41].

The target genes *HcAOC3* and *HcTAT* were examined to verify the expression stability of selected candidate reference genes in this study. Our LYD transcriptome sequencing study indicated that the two genes had high expression level in the root. However, there were conflicting results in the relative expression of *HcTAT* among different organs when *18S* was used for normalization. The relative expression of *HcAOC3* in root using *18S* also differed significantly when the most stable reference genes (*ACT*, *60S*, and *ACT*+*60S*) were used for normalization. Our result clearly suggested that the utilization of unstably expressed reference gene without validation may lead to biased results [42].

To summarize, from the present study, the combination of *60S*, *TUB*, and *AP4* was appropriate for commercial flower buds of different landraces whereas *ACT* together with *60S* was suitable for normalization under different organs. In addition, *AP4* exhibited the most stable expression in flower buds among different developmental stages. The relative expression analysis of target genes *HcAOC3* and *HcTAT* confirmed the correctness of these results using four statistical software programs. The stable reference genes identified in the current study collectively laid a solid foundation to analyze gene expression in *Hemerocallis citrina* Baroni. with RT-qPCR.

## Supporting information

S1 Fig Flowers of three different landraces. (TIF)

S2 Fig Standard curves of the six candidate reference genes. (TIF)

S3 Fig Specific PCR product for each gene were shown by 1.5% agarose gel electrophoresis. (TIF)

S4 Fig Melt curves of six candidate reference genes showing single peak. (TIF)

S1 Table Expression stability values for the six candidate reference genes in the LYD samples as calculated using the NormFinder algorithm. (PDF)

S2 Table Standard Deviation (SD) for the six candidate reference genes in the LYD samples as calculated using the BestKeeper algorithm. (PDF)

S1 Text The sequencing data of two target genes *HcAOC3* and *HcTAT*. (PDF)
