# Supplementary figures and images for: Identification and validation of reference genes for quantitative real-time PCR studies in long yellow daylily, Hemerocallis citrina Borani
Source: PLoS One. 2017 Mar 31;12(3):e0174933. doi: 10.1371/journal.pone.0174933 (PMC5376306; doi:10.1371/journal.pone.0174933)

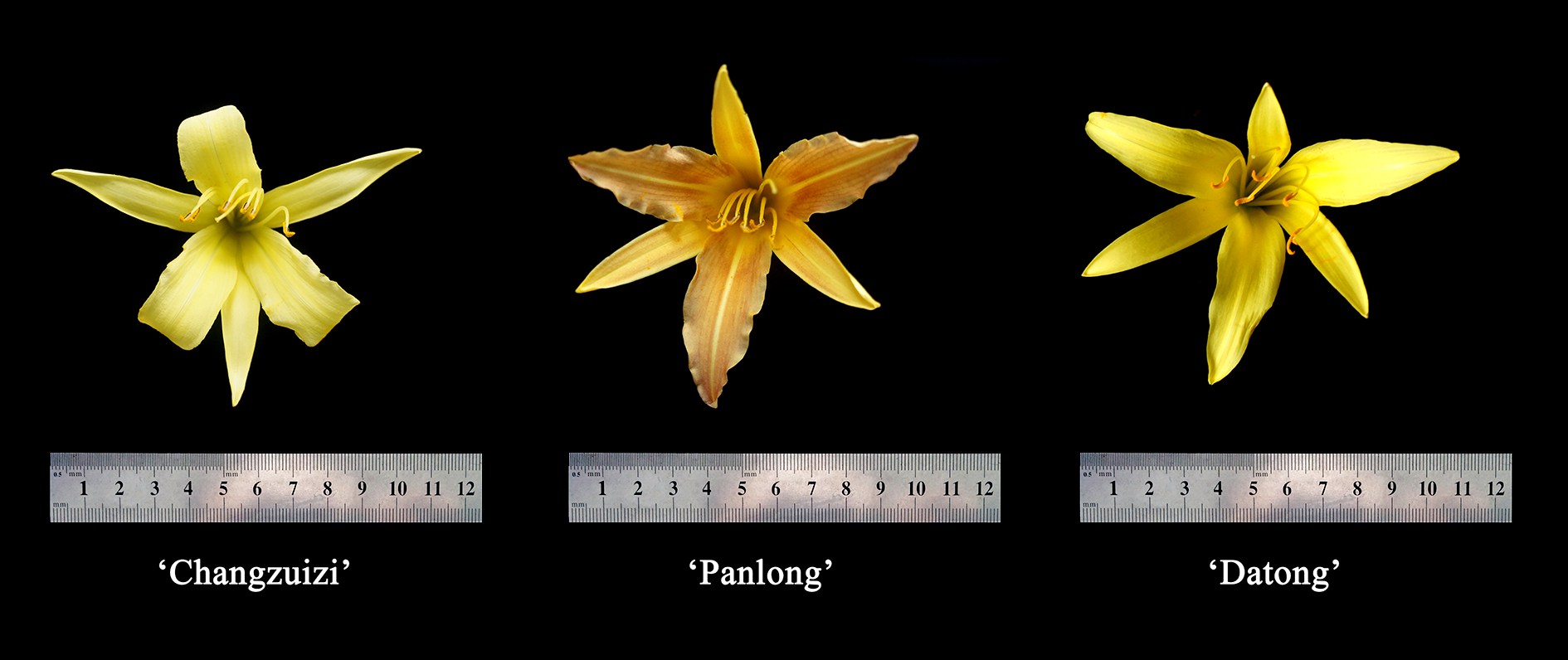

Supplement: S1 Fig — (TIF) [file pone.0174933.s001.tif]

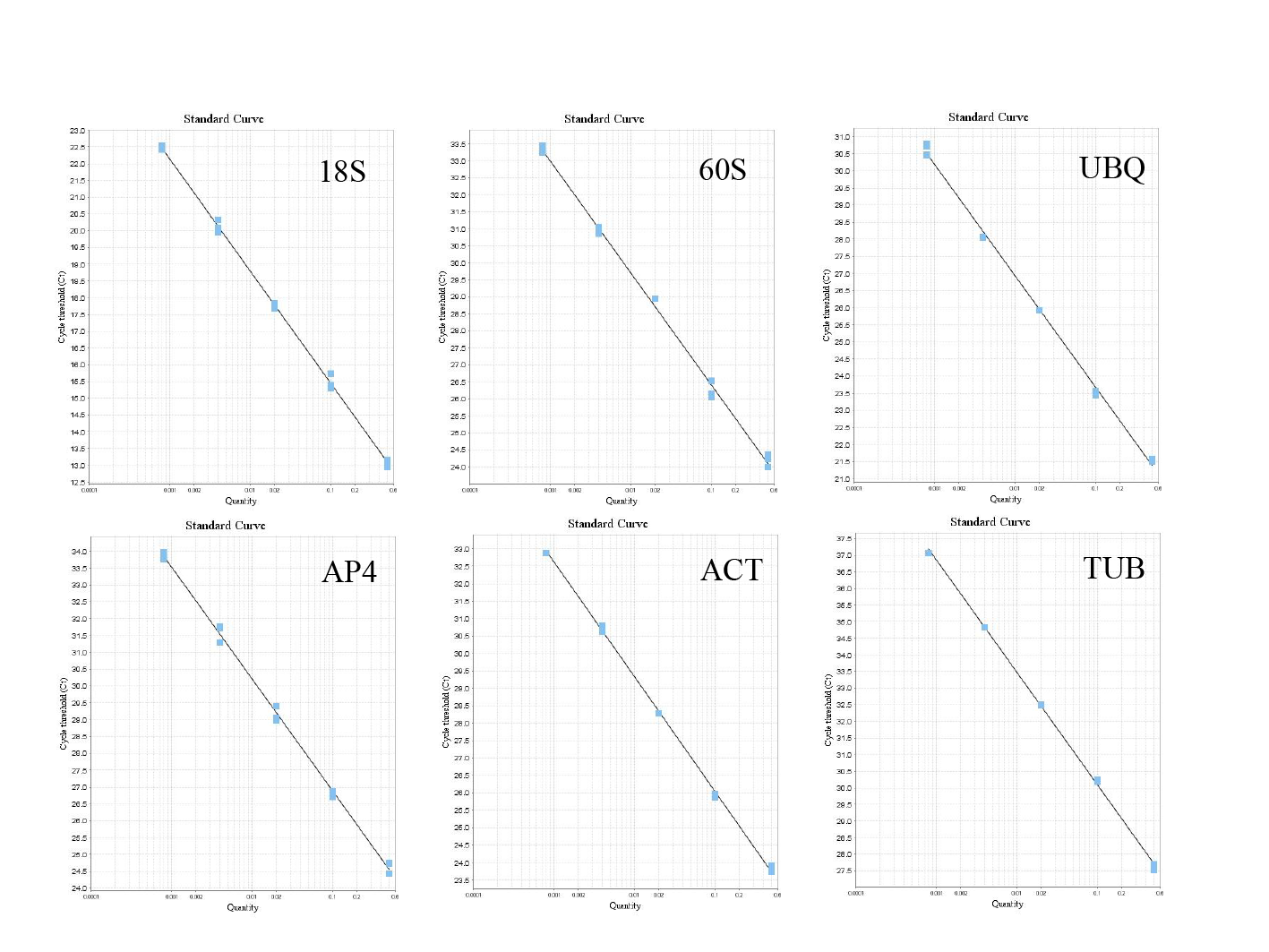

Supplement: S2 Fig — (TIF) [file pone.0174933.s002.tif]

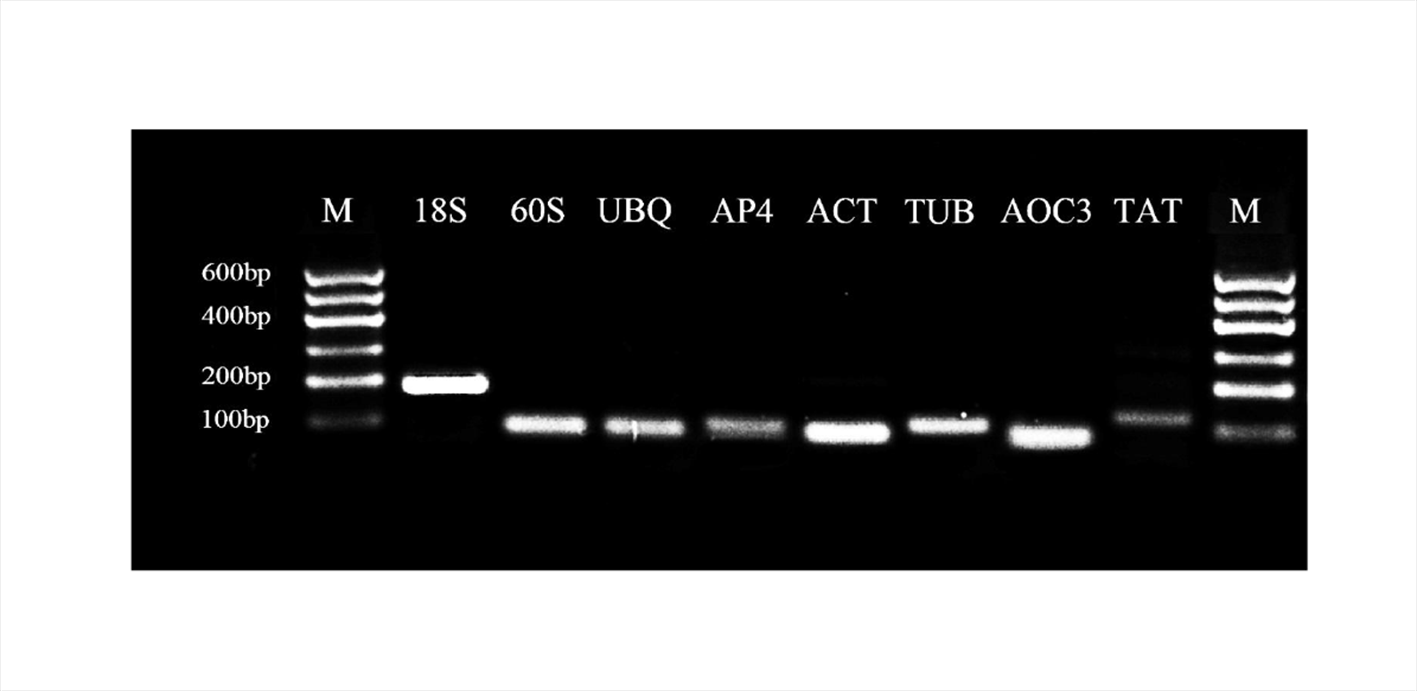

Supplement: S3 Fig — (TIF) [file pone.0174933.s003.tif]

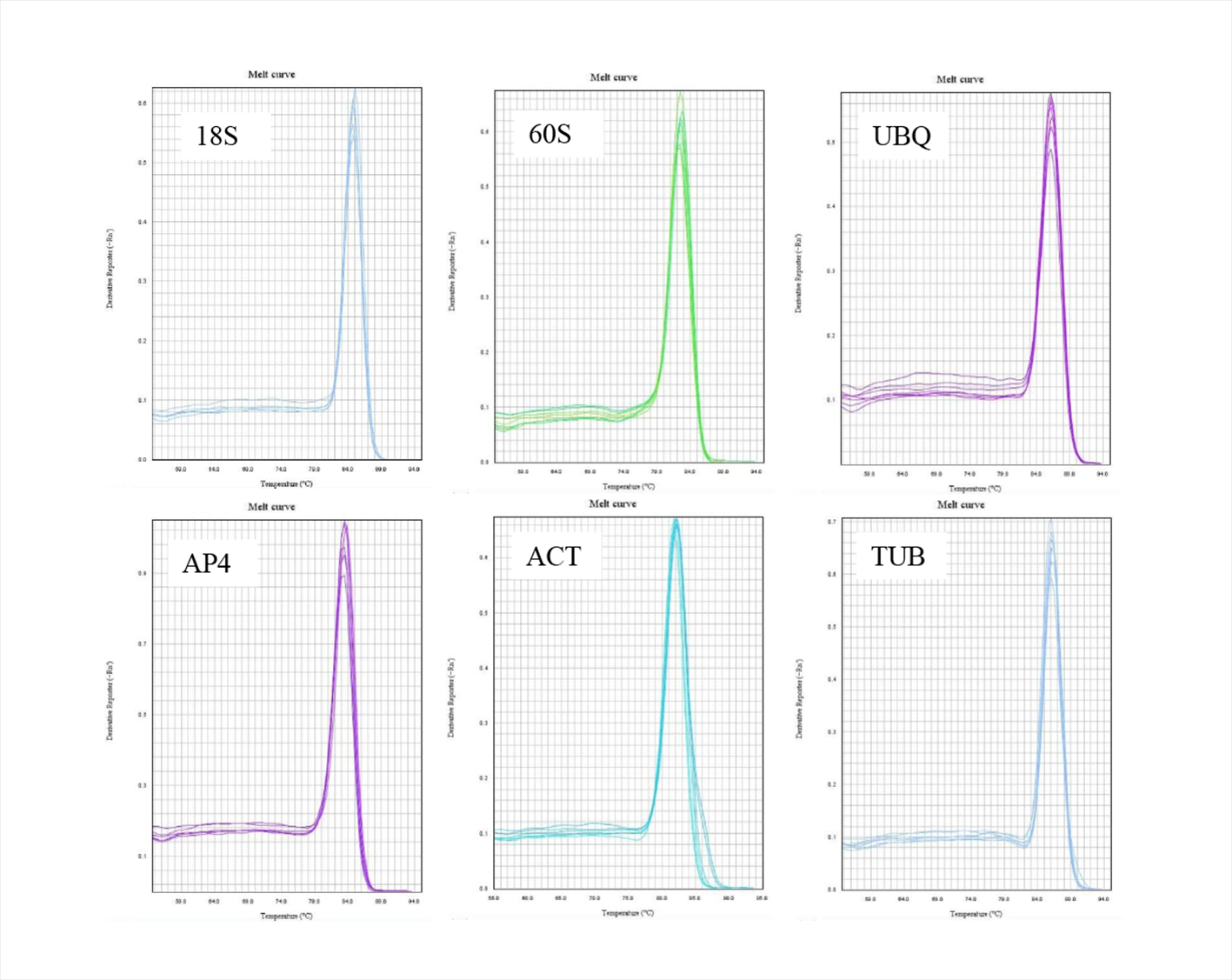

Supplement: S4 Fig — (TIF) [file pone.0174933.s004.tif]
