# Supplementary material for: Identification and validation of reference genes for quantitative real-time PCR studies in long yellow daylily, Hemerocallis citrina Borani
Source: PLoS One. 2017 Mar 31;12(3):e0174933. doi: 10.1371/journal.pone.0174933 (PMC5376306; doi:10.1371/journal.pone.0174933)
Supplement: S1 Table — (PDF) [file pone.0174933.s005.pdf]

S1 Table

Expression stability values for the six candidate reference genes in the LYD samples as calculated using the NormFinder algorithm.

Among flower buds at different developmental stages ('Datong')		Among different organs ('Datong')		Among commercial flower buds of different landraces		All samples	
Ranking	Stability value	Ranking	Stability value	Ranking	Stability value	Ranking	Stability value
AP4	0.180	*ACT*	0.044	*60S*	0.055	*AP4*	0.112
TUB	0.198	*UBQ*	0.072	*TUB*	0.085	*UBQ*	0.137
ACT	0.223	*60S*	0.073	*ACT*	0.087	*TUB*	0.150
18S	0.241	*TUB*	0.088	*AP4*	0.092	*60S*	0.155
UBQ	0.251	*AP4*	0.108	*18S*	0.164	*ACT*	0.164
60S	0.284	*18S*	0.139	*UBQ*	0.191	*18S*	0.177
