# Supplementary material for: Identification and validation of reference genes for quantitative real-time PCR studies in long yellow daylily, Hemerocallis citrina Borani
Source: PLoS One. 2017 Mar 31;12(3):e0174933. doi: 10.1371/journal.pone.0174933 (PMC5376306; doi:10.1371/journal.pone.0174933)
Supplement: S2 Table — (PDF) [file pone.0174933.s006.pdf]

S2 Table

Standard deviation (SD) for the six candidate reference genes in the LYD samples as calculated using the BestKeeper algorithm.

Among flower buds at different developmental stages (‘Datong’)		Among different organs (‘Datong’)		Among commercial flower buds of different landraces		All samples	
Ranking	SD	Ranking	SD	Ranking	SD	Ranking	SD
18S	0.64	*ACT*	0.43	*60S*	0.53	*TUB*	0.62
AP4	0.66	*60S*	0.46	*TUB*	0.63	*ACT*	0.71
TUB	0.68	*UBQ*	0.68	*UBQ*	0.63	*AP4*	0.81
ACT	0.77	*18S*	0.71	*AP4*	0.83	*18S*	0.96
60S	1.06	*TUB*	0.79	*18S*	0.84	*60S*	1.17
UBQ	1.23	*AP4*	1.03	*ACT*	0.84	*UBQ*	1.41
