# Supplementary material for: Identification and validation of reference genes for quantitative real-time PCR studies in long yellow daylily, Hemerocallis citrina Borani
Source: PLoS One. 2017 Mar 31;12(3):e0174933. doi: 10.1371/journal.pone.0174933 (PMC5376306; doi:10.1371/journal.pone.0174933)
Supplement: S1 Text — (PDF) [file pone.0174933.s007.pdf]

The sequencing data of *HcAOC3*:

GTAATATTCAATTAACTCTGAGAAATGTGTAACCATTAAACTGATAAACTGTCAGAATAT
CGAAAATGTTCATAATGAAATCATCACATAGAGAATTTAGAAGCAAAACAACAGAAAA
AGTCATAACCGGGTGACATTGTAAGGACTGATCCAGAATCAGCTAAAAAACTGTTTATA
ACATCAGCCATTTATTTTTGCAAGAAATGGGCAAACCATAACCATCCTGAAGGAGATGG
GAGTTATAAAGTACAGCCTTATTTAACTTTGGAAGTTCGAGTTGCCATTAGTTTTCTTCA
ATGCTTACAACAGTGATTTCAAAGCTTCGCTACCAAACCATTAGGAATTGACTTTGAAG
TGCCATTCTCCTTCAGCTCAATTTCATTACTACTGGGAGGAACGTCCACTGCTGGAGAA
CAGTTGAAAAAACCATGAGGCATGAGCATGAAACCTGTGCGTTCCACAGGCATAACTG
GCCAATCTTCCAGCCGAGGAATATGAGTGACTCCAAACACGTACCAGAGAACAATGTT
AGTCTCCTCAAGTGATCTGTTCTTCTTAACCCATGTAGCTAATCCCTCATTCACACGTGG
ATTTTGATTGGGAAATTCTCCACCTGGGAATATCTCATCACGTTTGTATTGCGTGACCCA
AAGGTTGTGCTTGAGAAAGGCTGCCCTTCTCAGAAAGGCTGCCTCTGGACTAGCCAAT
GGTAGGCAGTTAGAACCAGGAACAAGCTTATAACCTGTAAGCTGTCCTGTTCGATTAA
CAGTTCTGGTATTCCTTACAATCCAATGGCGTGCAGCAAGAGGATCACAGTCACGCATT
GCTTGTAGTTCAGACTTAAGGAGCTTCTCTTCAGCATAGAATGCATTATTATGAACATTA
CTTTGGCCTGGCTTTTCAACTTTAACATTCACCTCAACCACCTGATTGAAGGTTTCATT
GGGCTTACAATCAACAGCCATGTCCATACGAGCAACAAAAAAATGTTGATGGACTGGA
GCATATAAACCTGGAGCAATTGTAGTTCCATATTTTCTTGATTCTCCAGGAGGCAATGCT
CCCAAACTGAGAATCCCAGTAAGTTTTACTTCTGCTTCAATTTTACCGTCCTGATAGAA
ATGCCAAAAAAAGCCATATTCGTAGTTAGCCACCGTACATATAAAAGAAACGGTCAGC
CTTCTTGAGCGTCGTACCTCTGCTAATCCTGTTCTCCAATCTTGATGCTTCCACAATATT
CCATGATCCTCTTCGTGCAAGCAAACACAATTTTCAATAGTCTCTACACCCCCAGTGTA
ATTAGTAAAATGTGCATCGAAATATTTTATGTAGCCTAGACAATCGCATCCCTTTTTAAG
AGAGTGAGCATTTTTTCCAAGCCCATCTTCCCCAGCATCAAAAGCATTCTTACGGTAAT
GAGGTTCATCTGGATCTCCATAAGGCACTACCATTTCAACAAAACTCAACCTGTGAGCT
ACAGGTCTGCGACCACGGCTGCCATCGATATATGCTACAGAATATATAACCAATCCCTCT
CTGGGAGTGAACCCGATTCGAAAATTCCACTTCTGCCATTCGATAAAATGACCATTCAC
ACGAAAGCTTGGGCCATCAGGCTGCAGAATTTGCAAAGATTTCACATCACTACGATCA
ACACCTCCTCTTGTATCTCCTGGAGTGTAGTTTCTTAACGGATCTGCTGGAGGAAGAGG
AACCAGCTTCCTATCTTCGAACTCAACGATAACCACATTTTGGATATCAACAAGAACAT
AGATGCCCTCAACGGGGCGTGCATAACCATTCTCCATAGGACAGTCACTCTCTGTCCGA
CAAAAAATCAGTGGTTTAGCAAGTCGTCGGCTAGGAGAATCAGCTTCACTGTGATAAC
CAACACACCAGGCATCTACCATCACCAGATCCATATCCTCTATGCCTCGCCTCTTCATTG
CTTCTTTGAATGGAGGATAATCTTTTACAACAGCTTCACATTCTGCATATTCCACAGCAT
CCATTGGCGGTTGTACATCAGGTACAACTTCTGATGAAATGACCTTCCCCCTATGATGA
CCGCCACGAGTTGCAGCATGTACCTCAGTCAGCTCTACAATCCATATGCTCGTCTCATTT
GATTTCTTGTTATAGACAACCAGCCTAGCCCTCCTCGGAGGTAACTTGCTAGGAATAAT
GGGTCCACCTTTTGTTCTGGGTACCAGTGATGGTTGGAAGGGAGGGAAAAAATATGCA
TCAGCCAGAGCAACGATATTCTTCTCAGGTTCCAATAGAACCACTTCAACAAAGCGCA
TGCTATCTCTAACCTCTGGAGTTGCCCCCGCAGCCCTGACTGTTCCTACTGCCACAGCG
ATTTCGGCAGCAGATAATGGATCTAAAGGGTGGCTTGATTGAGCCCTCATCATCACTGG
GATACCCTTCGGTGAAGCCTTGGTGGTCGGAGCCGGATCTTGAAGATCATCAACCGGC
GTGATCAGGGTAGCCACAGCCGTCGATCTCTTGCCGCCACTCCCTCCATCTACCGTCCA

TTCCTGGACCAAACCGGAACCTCCGACATCCTTGGATTTGGCAGCAACAATCCTGACC
TTAGGGTTAGGGTTTGGAACGCAGCAAGGCGTCGCTTTTTCCGAAGTTGCGGCCATTG
AAGAGGGTTTGCGAGCTCACCACCGAATCCACCGTAGTTTAGTAAGTCTCCCTCCTCA
CAACGATCTGACATACAGCAGATCTCCGTTTCCTTGTTTTCACCTCGGTCCCCTCCAAA
ACCCTAGAAGGCCCCCCTTCCCCCCCGCTAAAAATCCCGCCTTTTTAAGGATAAAGATC
GCACCTTCTTGGGGATGGTGAGGAGAGGGGGGGTTTGAAAAGATAGCAACTTTGGTG
GGTTTGTCGTTCGCCGTCAAGAAAGGAAATAGAGAGAGATTGAAGGGAAAGGATTGC
GAGAGAGAAAAAGGTAAGAAAGAAACAAAAATGGCAATGTCGAATATTTAAGAGTCG
GAGGAAGGGGGAGGGTAGTAAGAGAGAGAGAGAGAG

The sequencing data of *HcTAT*:

AGATGTCCCAGTTTTATACTCCAGAGGCAAAGAGTTTGTCAACCCACTGGCACTGACC
ATAGATCATCCTCCGAAATACTTCTTTCGCAATGGACAACGGAGGAACCAACAAGAAG
TGGAACTTCGAGATCTCCATCGACCCAGCGGGCGGCGGTGGCGCGTTCTCGGTGCGAG
TGTATCTCAACAGCCTGTTCGCTCAAATCGATAAAACCAATGGCAGAACTGTAATTCCT
CTGGGACATGGCGACCCTTCCAGTTTCGAGTGCTTTCGTACGACTACCGTAGCTGAGG
ATGCCGTTGAGGCTGCTCTTCGGTCGGCCGAGTTCAACGGGTATTCGCCCGCCGCCGG
CCTTCCATCTGCTAGAGCTGCAATTGCAGAGTACCTCAATCAAGATCTTCCCTACAAGA
TATCTCCTGAAAATGTATATGTTACTGGTGGCTGCTTTCAAGCAGTCGAGATCATTATCT
CGGTTCTTGCACGTCCAGGTGCAAATATATTGTTCCCAAGGCCTAACTTTCCTCTATATG
ACGCACGAGCTGCTTTCAGTAAATTAGAGGCTCGTCATTTCAACCTCATCCCTGAGAG
GGGGTGGGAGGTTGACCTAGATAATGTTGAGGCTCTTGCAGATGAGAATACTGTTGCC
ATGGTCATAATTAACCCGGGGAATCCTTGTGGGAGCGTGTACACTTATCAACATTTGTC
CAATATTGCTGAGACTGCAAAGAAGCTTGGCATAATGGTAATAGCAGATGAAGTGTACC
ACCATTTGACCTTTGGGAGCAATCCTTTTGTGCCAATGGGAGTTTTTGCTCATATTGCTC
CTGTTATCACTCTGGGGTCTATATCAAAGAGATGGTTAGTTCCTGGTTGGAGACTTGGG
TGGATGGTAACAAATGATCCCAATGGCATTCTTAAACAAACTAAGATTGCTGAGAGCAT
TGAAAGCATTTGCAATATCACAACTGATCCGGCAACCTTTATTCAGGCAGCAGTTCCTC
ACATCATCAAGAGCACAAATTATGATTTCTTCAAGAAGACCATTGATATACTAAGTCAAT
TATCAGATATATGTGATGGCAAAATAAAGGAGATTGACTGCATTACTCTTCCACATAAAC
CAGAAGGATCCATGTTTGTAATGGTAAAGTTGAATTTGTCTGATCTGGAGGACATTACT
GATTGTTTTGACTTCTGCCTCAAGTTGGCCGAAGAGGAGTCCGTGATAGTTTTGCCCGG
AAGGGCTGTGGGATTGGCAGATTGGCTTCGCATCACTTTTGCCATCGACCCTTCCTCCC
TCGAGCTAGCATTTGACAGGATTAAAGCCTTCTGCCAGAGGCATGCAAAGCTCAATCA
GTAACAAAACCGTTGGTATAATAAGTACGTTGGTGATGATCGGCAACTATGCGTTGGAT
CTCACTCTTATTTGTTCAGACTCCATATATAATTGGTGAAGATCAACCAACACCGCGTTG
GATCTCACCTTGTCTTGTCATTTGCTTAAGTTGGTGGTGATCAAGCAACATAATGTTATG
CTGGATCTCACCTCTTCTTCGTTGTTGTTTTCTTGTTCACAGTACATTTTCATAATAGTAT
GTGGTTCATGCTGTTAAAAGTATGGTTGTTATATTGGAGAAATCTG
